# Are Residents' Extended Shifts Associated With Adverse Events?

**DOI:** 10.1371/journal.pmed.0030497

**Published:** 2006-12-12

**Authors:** Mariana Szklo-Coxe

## Abstract

Szklo-Coxe discusses a new prospective study in PLoS Medicine that examined the relationship between residents' work patterns and adverse clinical events.

Heated debates and controversies surrounding the regulation of medical residents' work hours have raged for over twenty years. In the wake of Libby Zion's untimely death in 1984 and resulting recommendations by the Bell Commission, New York State enacted legislation (Code 405.4) governing residents' working conditions and supervision [1]. Since then, there has been growing interest in regulating residents' work hours, culminating recently (2003–2004) in national guidelines and legislation on duty-hour restrictions both in the United States and Europe [2,3].

These regulations continue to be the subject of intense debate and bitter controversy, yet at the same time, the medical profession has become increasingly aware of the complexities of balancing restricted work hours with resident education, well-being, and the profession's key priority—quality of care [4,5]. However, there is still a scarcity of research on the relationship between residents' work schedules and adverse events (AEs) [4,6], defined as injuries due to medical management rather than the underlying condition of the patient [7]. This lack of research is in striking contrast to the wealth of research on the relationship between work schedules and adverse events in industrial and transportation settings [8] (long work hours, for example, were implicated in the catastrophic Exxon Valdez oil spill [9]). In 2000, the Institute of Medicine reported medical errors to be a leading cause of death in the US [10], responsible for 44,000–98,000 in-hospital deaths [7,10,11].

To date, epidemiologic studies, including those upon which the Institute of Medicine's extrapolations were based, have focused on estimating the incidence of adverse events and their consequences—namely, the magnitude of harm resulting from AEs in terms of morbidity (such as patient disability) and mortality [7,10,11]. A benchmark study of New York State hospitals found that 70.5% of AEs produced short-term disability in patients, 2.6% led to permanently disabling injuries, and 13.6% resulted in death [7]. Although the relationships of adverse events to specific provider types/locations and to negligence have been examined [7,11]—with 27.6% of AEs attributed to negligence [7]—the epidemiology of AEs has not been fully clarified.

A new study in *PLoS Medicine*, by Barger and colleagues [12], investigates the contribution of work-hour organization—specifically interns' extended shifts—to adverse events. This relationship is of interest because it could inform future guidelines on residents' working schedules; it could also help hospitals to adopt new strategies for complying with current legislation on restricting work hours and to weigh the costs and benefits of such strategies.

## The New Study

Prior studies have focused on patient outcomes before and after mandated restrictions of residents' work hours or, in randomized studies, on patient outcomes upon introduction of schedules designed to reduce work hours. These studies had varying findings: some studies found that restricting work hours was associated with an increase in the rate of inhospital complications and adverse events or errors [4,6], some found no change in the rate, and some found a reduced rate [13,14,15]. In their study, Barger and colleagues shifted the focus beyond just work-hour limitations to the specific way in which work was organized—namely the frequency of extended shifts in the month. There has been little research on the relationship between work organization—such as extended shifts or on-call days—and medical near-errors/errors [13,16,17], and an even greater dearth of research to date on preventable adverse events (an adverse event attributable to error), with one study on cross-coverage a notable exception [6].

Landrigan and colleagues had previously shown, in a randomized controlled trial, that eliminating extended shifts led to a decrease in medical errors, but the study was underpowered to examine adverse events [13]. Barger and colleagues therefore attempted to clarify the relationship between extended residency shifts (those that lasted 24 hours or more) and adverse events resulting from medical errors attributed to fatigue or sleep deprivation. They conducted a prospective, nationwide Web-based survey in the US in which 2,737 residents in their first postgraduate year (interns) completed 17,003 monthly reports. The cross-sectional associations between the number of extended duration shifts worked in the month and the reporting of attentional failures, as well as of fatigue-related significant medical errors and of consequent preventable adverse events were assessed by considering each subject as his/her own control and calculating pooled odds ratios. These odds ratios estimated the odds of having one or more attentional failure(s); the odds of having one or more fatigue-related medical error(s); and the odds of one or more adverse event(s) resulting from fatigue-related medical error(s), with respect to frequency of extended shifts per month.

Barger and colleagues found that fatigue-related medical errors were increased 3.5-fold and 7.5-fold, respectively, with one to four and with five or more extended shifts in a month. The respective odds ratios for fatigue-related adverse events were 8.7 (95% CI, 3.4–22) and 7.0 (95% CI, 4.3–11), respectively. Interns working more than five extended duration shifts per month reported more attentional failures during lectures, rounds, and clinical activities than interns working fewer extended shifts.

## Extended Shifts Compromise Patient Safety

Barger and colleagues' study advances research on adverse events and exposes interns' extended shifts as a weakness in the design of the residency system. Extended shifts were related to increased fatigue-related adverse events even among those interns (80% of the sample) well within compliance (mean work hours of 65 hours) of the Accreditation Council for Graduate Medical Education weekly work limits of 80 hours [2]. By uncovering an association between adverse events and extended shifts—even one to four per month—the study unmasks “latent errors” [18] that are threatening safety in complex systems [10]. Restrictions on extended shifts, not just weekly duty-hours, should be considered when designing residents' schedules. This present new study can help inform a more comprehensive approach to patient safety, as called for by the Institute of Medicine [10]. The findings are consistent with prior research on specific work-hour organization factors and medical near-errors/errors [13,16,17].

## Limitations of the Study and Future Directions

Nevertheless, the study also has weaknesses, including concerns about possible biases. Landrigan and colleagues considered sleep deprivation to be the likely explanation for the link between extended shifts and medical errors [13], and yet Barger and colleagues, rather than examining sleep deprivation as an independent variable in their study, embedded sleep deprivation in their definition of medical errors and the follow-up questions regarding adverse patient outcomes and fatalities. Only asking interns about medical errors caused by fatigue or sleep deprivation and not asking about errors unrelated to causality may have inflated the associations found. Interns may have systematically responded affirmatively to this item and been more likely to over-attribute their errors to fatigue/sleep deprivation, relative to medical errors unrelated to causality or to the inverse question which did not explicitly present specific causes (“Do you believe you made any significant medical errors other than due to sleep deprivation or fatigue?”). Inquiring about adverse events irrespective of medical errors might also have been useful, as interns may not have recalled or recognized that a medical error preceded the event [19]. As significant medical errors and resulting adverse events were not defined, interns' interpretations may have varied; for example, some may have defined serious errors very conservatively just as those ending in fatalities. In addition, types of errors (for example, errors in diagnosis or medication) were not assessed and would be useful to include in follow-up studies to provide additional specific recommendations. Future studies should also extend the current findings by considering extended shifts in a broader context of various patient and work schedule factors such as: patient illness severity [6], patient length of stay, cross-coverage [4,6], distribution of rest hours [20], and inadequate supervision—which is often neglected relative to work hours despite being a critical component of the landmark New York regulations [1].

## Redesigning Residency Systems

Implementing alternative coverage schedules requires consideration of potentially dangerous trade-offs. For example, limiting work hours by using cross-coverage [4,6] or shift work [21] has been associated with detrimental impacts on residents' training experiences/satisfaction [21], loss of continuity of patient care, inhospital complications, and an increase in the probability of adverse events [4,6]. Lessons from interventions and proposed solutions (developed to comply with the Accreditation Council for Graduate Medical Education requirements and stringent European guidelines/legislation [2,3]) provide meaningful insights into work-hour redesign. For example, the United Kingdom's Academy of Medical Royal Colleges has recommended that ten junior doctors be used in a rota to provide 24/7 coverage and meet the UK's maximum of 56 weekly and 13 consecutive work hours [22].

## Reducing Adverse Events

Adverse events are a significant public health problem [10], and any solution will probably require a multilevel approach which considers behavioral and environmental factors [23]. For example, studies have shown that taking naps improved residents' memory [24], while a 14-hour shift [14] and other schedules [13] to minimize work hours decreased errors and maintained safety. Public health approaches—including behavior change theories for injury prevention [23], ecological approaches targeting individual and environmental factors [25], and injury prevention models focusing on adverse events/injuries, rather than medical errors [19]—are promising frameworks for understanding adverse events and their implications in the context of residency training. Applying complementary approaches from industrial medicine [20] and lessons from high-risk industries like aviation, to study near-miss events for example [26], might prove beneficial.

Recently, a unifying framework was proposed to study long work hours across industries [20]. More efforts to bridge approaches and increase collaboration across disciplines investigating adverse events/injuries are needed. Thoughtful applications of multidisciplinary frameworks may improve translation of findings into effective and sustainable recommendations to improve patient safety without damaging residency education.

## Abbreviations

AE: adverse event
